# Resistance to third-generation EGFR TKIs - what can be expected from the fourth-generation?

**DOI:** 10.20517/cdr.2026.79

**Published:** 2026-09-03

**Authors:** Wolfram C. M. Dempke, Klaus Fenchel, Loretta Sullivan, Martin Reck

**Affiliations:** ^1^Medical Clinic III, University of Munich, Munich D-81377, Germany.; ^2^Medical Oncology Department, MVZ Saalfeld, Saalfeld/Saale D-07318, Germany.; ^3^Immuneering Inc., Boston, MA 02142, USA.; ^4^Department of Thoracic Oncology, Airway Research Center North, German Center for Lung Research, Lungenclinic Grosshansdorf, Grosshansdorf D-22927, Germany.

**Keywords:** Non-small cell lung cancer, EGFR TKIs, third generation, resistance mechanisms, fourth generation

## Abstract

Tyrosine kinase inhibitors (TKIs) have significantly changed the treatment of non-small cell lung cancer (NSCLC) harbouring epidermal growth factor receptor (EGFR) mutations, and osimertinib is now established as first-line therapy for NSCLCs. Combination therapies (e.g., osimertininb plus chemotherapy; lazertinib plus amivantamab) have been shown to improve median progression-free survival (mPFS) and median overall survival (mOS) relative to monotherapy. However, due to several resistance mechanisms, patients experience disease progression following EGFR TKI treatment. On-target resistance mechanisms include additional mutations (e.g., C797S/G/N, L718Q, L844V, G724X). Within the heterogeneous group of off-target resistance mechanisms, human epidermal growth factor receptor 2 (HER2) amplifications, mesenchymal-epithelial transition factor (MET) alterations, oncogenic fusions (e.g., BRAF, FGFR, RET), histological changes, epithelial-mesenchymal transitions, and alterations of the RAS/MEK/ERK and the PI3K/AKT/mTOR signal transduction pathways are critical and can confer resistance to EGFR TKIs. Several drugs have been identified to inhibit these pathways, with some of them already approved for clinical use. Most fourth-generation EGFR TKIs are orally bioavailable and are mainly allosteric thiazole amide-based reversible inhibitors. Their activity results from selective binding to an allosteric site, which can alter the EGFR protein conformation, allowing them to bypass C797X. A recommendation for the optimal treatment strategy and sequence for NSCLC patients with acquired EGFR TKI-resistant tumours still cannot be given. An improved understanding of the underlying resistance mechanisms will help to pave the way for the development of innovative and highly specific drugs for the therapy of osimertinib-resistant NSCLCs. The putative clinical relevance of fourth-generation EGFR TKIs for NSCLC patients needs to be defined, and many development hurdles need to be cleared before victory can be declared.

## INTRODUCTION

Non-small cell lung cancer (NSCLC) accounts for 80%-85% of lung cancer cases and is one of the major reasons for cancer-related death. The discovery of oncogenic driver mutations that occur frequently in the epidermal growth factor receptor (*EGFR*) gene (Europe: 9%-16%, Asia: 27%-73%)^[1]^ and the development of specific tyrosine kinase inhibitors (TKIs) have changed the therapeutic management of NSCLC harbouring these mutations dramatically during the last decade (reviewed by^[1]^). Amongst these alterations, exon 19 deletions (del19) and a point mutation in exon 21 (L858R) (so-called “classical” or “common” mutations) account for up to 85%-90% of EGFR mutations, whereas uncommon mutations (e.g., exon 20 insertions, T790M, C797S, G719X, S768I, L861Q and others - prevalence 10%-15%) represent a very heterogeneous and complex group within the exons 18-21 of the *EGFR* gene with over a hundred alterations identified to date^[2]^.

Based on the underlying EGFR mutation detected in tissue or blood samples from NSCLC patients, orally available EGFR TKIs are now the standard of care, and osimertinib (a third-generation TKI) is the established first-line therapy for NSCLCs with these EGFR alterations^[1,2]^. Moreover, several lines of clinical research have recently provided significant evidence that combination therapies (e.g., osimertininb plus chemotherapy; lazertinib plus amivantamab) can improve median progression-free survival (mPFS) and median overall survival (mOS) relative to monotherapy [Table 1].

**Table 1:** Summary of results of major clinical trials in first-line NSCLC harbouring del19 and/or L858R EGFR mutations

**Trial (NCT)**	**Phase (*N*)**	**Design**	**Results**	**Ref.**
FLAURA (NCT02296125)	III (*N* = 556)	Osimertinib *vs.* Gefitinib or Erlotinib 1:1 randomisation	ORR: 80% *vs.* 76% mPFS: 18.9 *vs.* 10.2 months mOS: 38.6 *vs.* 31.8 months	Soria *et al.*^[3]^ Ramalingam *et al.*^[4]^
FLAURA-2 (NCT04035486)	III (*N* = 557)	Osimertinib plus CTX *vs.* Osimertinib 1:1 randomisation	ORR: 83% *vs.* 76% mPFS: 25.5 *vs.* 16.7 months mOS: 47.5 *vs.* 37.6 months	Planchard *et al.*^[5]^ Jänne *et al.*^[6]^
MARIPOSA (NCT04487080)	III (*N* = 1,074)	Amivantamab* plus Lazertinib *vs.* Osimertinib *vs.* Lazertinib 2:2:1 randomisation	ORR: 86% *vs.* 85% *vs.* NS mPFS: 23.7 *vs.* 16.6 *vs.* 18.5 months mOS: NR *vs.* 36.7 months *vs.* NS	Cho *et al.*^[7]^ Yang *et al.*^[8]^

*Amivantamab is a monoclonal antibody targeting EGFR and c-MET. NSCLC: Non-small cell lung cancer; del19: 19 deletions; EGFR: epidermal growth factor receptor; ORR: objective response rate; mPFS: median progression-free survival; CTX: chemotherapy (platinum plus pemetrexed); mOS: median overall survival; NS: not stated; NR: not reached (after > 3 years); c-MET: cellular mesenchymal-epithelial transition factor.

**Figure 1:**
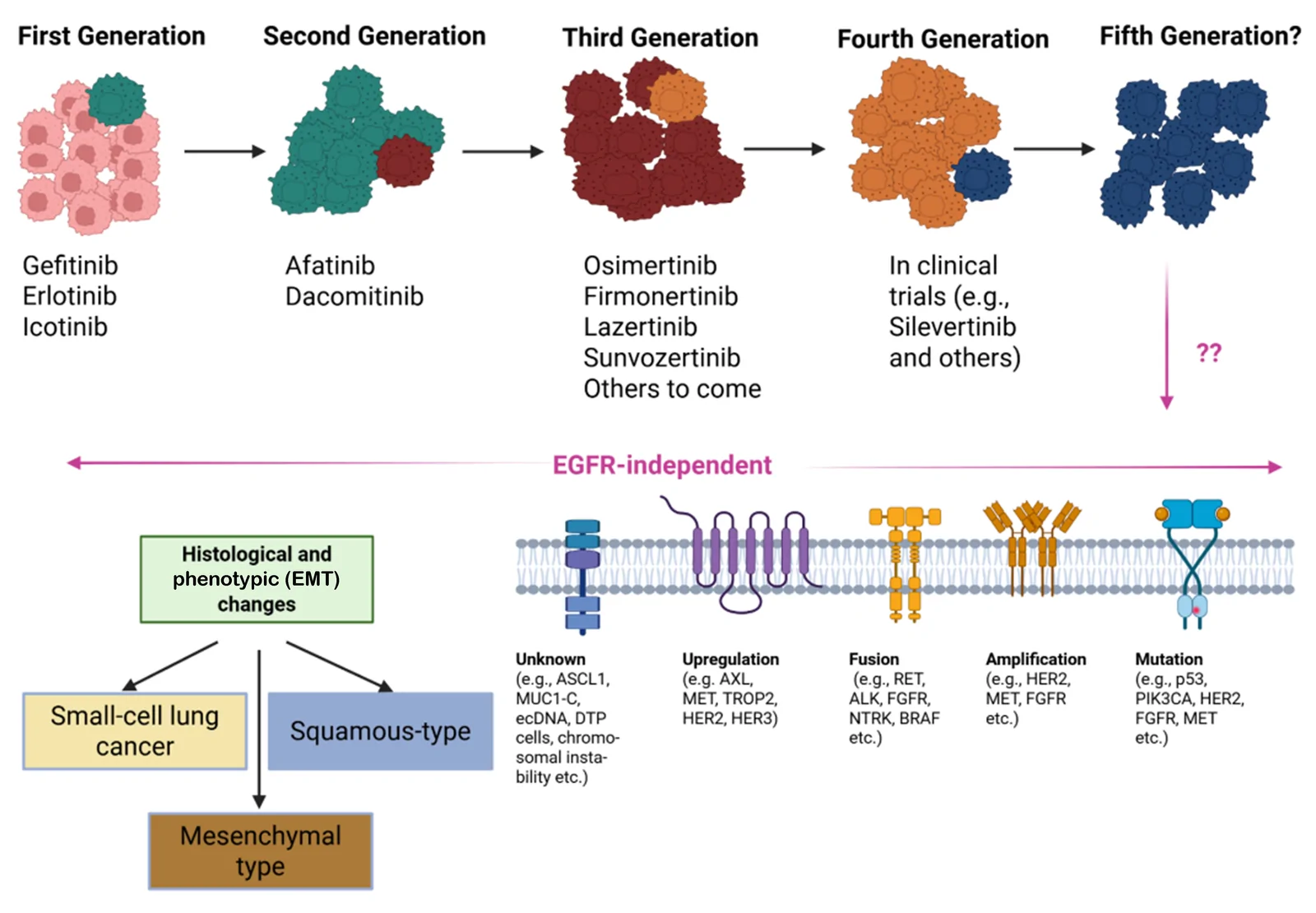
Development of different generations of EGFR-mut TKIs in NSCLC. EGFR-dependent resistance mechanisms are associated with different EGFR mutations (e.g., del19, L858R, T790M, C797S, and others). Currently approved TKIs are also listed. Fourth-generation TKIs targeting C797S are currently under clinical development. The bottom half of the illustration depicts EGFR-independent resistance mechanisms. ??: 5th-generation TKIs are currently unknown (no data in the literature so far). This figure was created using BioRender.com (modified after^[1,10-12]^) (https://biorender.com/o0zo69u). EGFR: Epidermal growth factor receptor; TKIs: tyrosine kinase inhibitors; NSCLC: non-small cell lung cancer; EMT: epithelial-mesenchymal transition; ASCL1: achaete-scute homolog 1; MUC1-C: Mucin-1-C; ecDNA: extrachromosomal DNA; DTP: drug-tolerant persister cell; AXL: AXL receptor tyrosine kinase; MET: mesenchymal-epithelial transition factor; TROP2: trophoblast cell-surface antigen 2; HER2/3: human epidermal growth factor receptor 2/3; RET: rearranged during transfection; ALK: anaplastic lymphoma kinase; FGFR: fibroblast growth factor receptor; NTRK: neurotrophic receptor tyrosine kinase; BRAF: B-Raf proto-oncogene, serine/threonine kinase; PIK3CA: phosphoinositide-3-kinase.

However, due to EGFR-dependent and EGFR-independent resistance mechanisms, patients experience disease progression following EGFR TKI treatment. This includes additional mutations (e.g., T790M, C797S, L718X), compound mutations (e.g., L858R/T790M/C797S)^[9]^, whereas EGFR-independent resistance mechanisms have also been identified [e.g., mesenchymal-epithelial transition factor (MET) or human epidermal growth factor receptor 2 (HER2) amplifications, fusion genes, RAS/MET bypass mechanisms, epithelial-mesenchymal transitions, histologic changes] [Figure 1].

For instance, the main mechanisms found in acquired osimertinib resistance were either the development of the C797S mutation or MET alterations^[10]^. Interestingly, the allelic relationship between T790M and C797S mutations can influence response to osimertinib^[11,12]^. Wang *et al.* have demonstrated for the first time that NSCLC patients harbouring concomitant T790M and C797S *in trans* (i.e., on different DNA strands) remain sensitive to a combination of gefitinib or erlotinib with osimertinib^[13]^. However, if these mutations are *in cis* (i.e., on the same DNA strand), no TKI alone or in combination was found to be active - a finding that adds weight to the proposal that allelic shifts of C797S from *in trans* to *in cis* may be indicative of a new resistance mechanism. Furthermore, this observation could pave the way for a more bespoke treatment option for osimertinib-resistant patients harbouring the C797S mutation, and it strongly underscores the need for a second biopsy following clinical progression after EGFR TKI therapy in NSCLC patients.

Overall, in more than half of NSCLC patients with metastatic disease and progression after osimertinib, the underlying resistance mechanisms cannot be identified, suggesting that non-mutational pathways may also play a significant role^[14]^. This adds weight to the demand for new EGFR TKIs (fourth generation) to treat patients with C797S and other resistance mutations in the near future.

## ACQUIRED RESISTANCE MECHANISMS

### On-target resistance mechanisms

**Figure 2:**
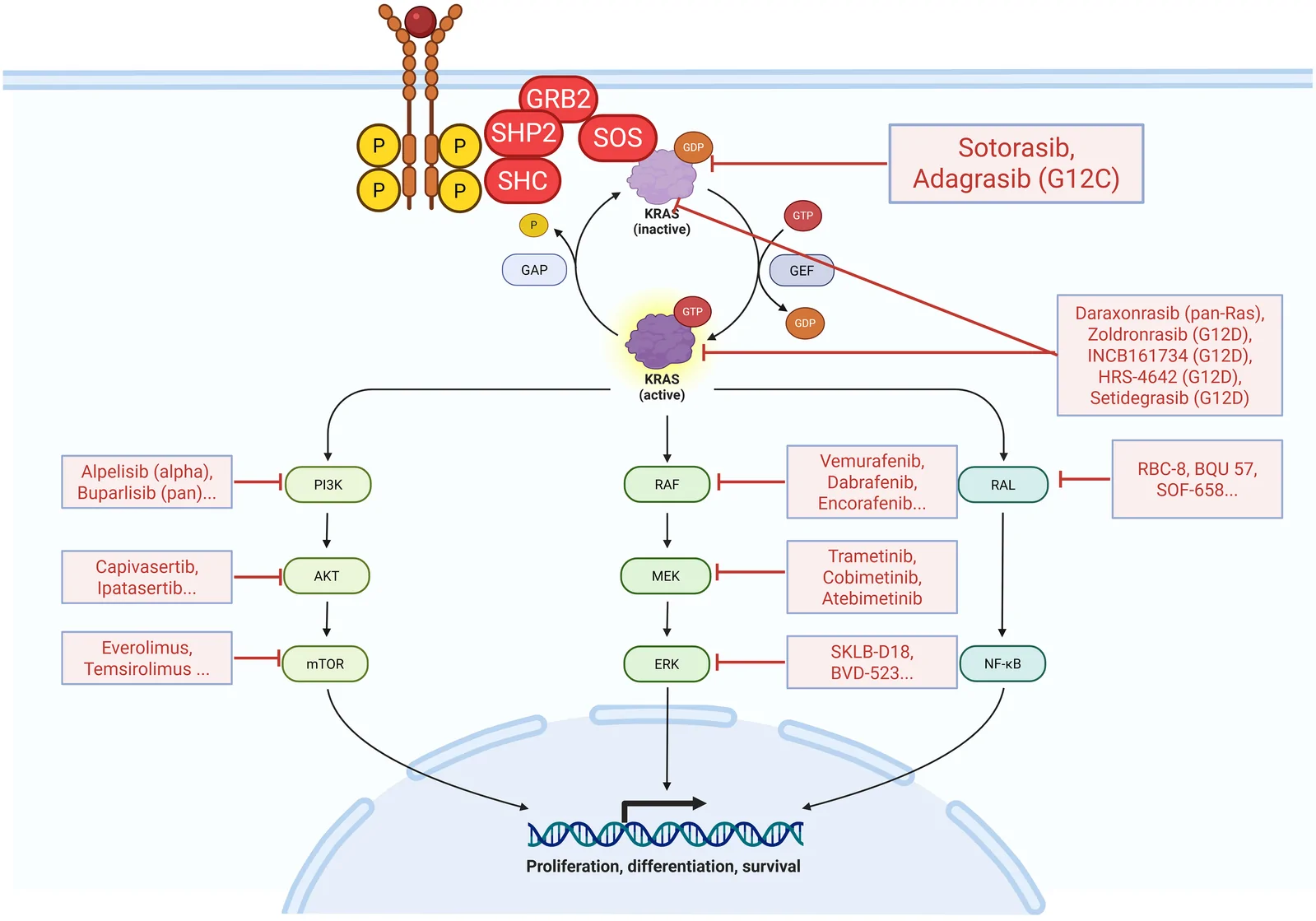
RAS and EGFR interactions represent a primary cell signalling axis that controls cell growth and division. EGFR is a cell-surface receptor that, when activated by its ligand, passes a signal downstream to RAS proteins, which then trigger the MAPK and PI3K cascades. The RAS signal transduction cascade has been implicated in EGFR TKI resistance, and several drugs have been identified to inhibit this pathway, with some already approved for clinical use. As of March 2026, 73 different RAS inhibitors have entered 236 clinical trials, essentially evaluating almost all RAS mutations. However, resistance develops rapidly, and toxicity remains a significant problem (e.g., daraxonrasib). In contrast, novel deep cyclic MEK inhibitors (e.g., atebimetinib) were found to be less toxic with comparable efficacy. Targeting ERK1,2 was a major challenge in the past as these inhibitors appeared to be very toxic in animal models; however, the newly developed compound SKLB-D18 (IC_50_ values: 38.7 nM for ERK1, 40.1 nM for ERK2, and 59.7 nM for ERK5) has shown a very favourable toxicity profile in preclinical models^[77]^. This figure was created using BioRender.com (https://biorender.com/xc5lpem). RAS: Rat sarcoma virus; EGFR: epidermal growth factor receptor; MAPK: mitogen-activated protein kinase; PI3K: phosphoinositide 3-kinase; TKI: tyrosine kinase inhibitor; MEK: mitogen-activated protein kinase kinase; ERK: extracellular signal-regulated kinase; GRB2: growth factor receptor-bound protein 2; SHP2: src homology region 2 domain-containing phosphatase-2; SOS: son-of-sevenless; SHC: src homology 2 domain containing; KRAS: Kirsten rat sarcoma virus; GDP: guanosine diphosphate; GAP: GTPase-activating protein; GTP: guanosine triphosphate; GEF: guanine nucleotide exchange factor; AKT: protein kinase B; mTOR: mechanistic target of rapamycin; RAF: RAF family of serine/threonine protein kinases; RAL: RAS-related proteins (RAL-A and RAL-B); NF-κB: nuclear factor kappa B.

Within the molecular structure of the EGFR, a conserved adenosine triphosphate (ATP)-binding site is located between the N-lobe and C-lobe of the kinase domain, which are connected by the hinge (amino acids 790-797). In addition, the ATP adenosine ring forms hydrogen bonds with amino acids at positions Q791 and M793, which appear to be of great importance for the conformational changes that, in turn, to enable ATP binding and catalysis^[15]^. A threonine at residue 790 within the hinge N-terminal region acts as the gatekeeper (regulatory function) and thereby controls the access of a given TKI to the active kinase site, while the C-terminal cysteine (at residue 797) is most commonly targeted by covalent inhibitors. A conserved α-helix is held in the “out” position by a flexible activation loop (A loop) during the inactive kinase state. Binding of EGF (ligand) to the extracellular part of the receptor leads to EGFR homo- and hetero-dimerisation, which then results in conformational changes within the kinase domain. These changes involve an inward rotation of the α-helix followed by the formation of weak H-bonds between amino acids E762 and K745 (so-called K-E salt bridge, necessary for the catalytic activity)^[15]^. Finally, following the enzyme-induced hydrolysis of the γ-phosphate of ATP, EGFR phosphoryl-transferase enzymes are activated, which then modify alcohol-containing amino acid side chains of the C-terminal tail of other tyrosine-kinase receptors^[16]^ [docking stations for other proteins such as src homology 2 domain containing (SHC) - Figure 2].

Several lines of preclinical and clinical research have provided evidence that the main cause of resistance to first- and second-generation EGFR TKIs is the development of the T790M mutation. Further research has also shown that T790M is associated with exon 20 insertions^[17]^. T790M is a missense mutation within the *EGFR* gene. It substitutes threonine (T) with methionine (M) at residue 790 of exon 20 of EGFR^[11]^, and the mutation blocks the EGFR TKI from accessing the ATP-binding pocket.

Furthermore, some evidence suggests that the T790M mutation can restore EGFR wild-type properties, preventing ATP-competitive TKIs from binding^[18]^. Moreover, several studies have demonstrated that the T790M mutation enhances the ATP affinity within the EGFR ATP-binding pocket and introduces steric hindrance that impairs binding of first-generation TKIs, thereby contributing to resistance^[19]^.

To overcome T790M-mediated resistance, third-generation EGFR TKIs have been developed and are now the standard of care for NSCLC patients harbouring EGFR mutations. In third-generation TKIs, the commonly used quinazoline backbone (first- and second-generation) has been replaced by an aminopyrimidine skeleton, which is very potent at blocking the ATP-binding site of the L858R and T790M mutations; however, it has weaker activity against del19^[20]^.

Despite these advances, a significant proportion of NSCLC patients harbouring EGFR mutations develop resistance to third-generation TKIs such as osimertinib or are osimertinib-refractory (FLAURA-2 trial: 17%)^[5,6]^. When osimertinib is used as first-line treatment for EGFR-mutated NSCLC, acquired resistance develops through heterogeneous on- and off-target mechanisms. The most frequent specific resistance drivers identified are c-MET amplifications (occurring in 15%-19% of patients) and EGFR C797S mutations (approximately 6%-24% of patients)^[10,21]^.

However, in the majority of these patients, the observed resistance appeared to be multifactorial (or unknown); additional mutations have also been identified, including C797S/G/N, L718Q, L844V, G724X, and others^[21]^. In this regard, much emphasis has been placed on the C797S alteration. This mutation significantly reduces the sensitivity of osimertinib to cysteine at position 797 (up to 1,000-fold), because serine lacks the sulfhydryl group. As a result, osimertinib loses its covalent anchor point, allowing ATP to outcompete the drug, which then results in the development of resistance^[22,23]^.

### Off-target resistance mechanisms

Within the heterogeneous group of off-target resistance mechanisms, HER2 amplifications and c-MET alterations (mutations and amplifications) have been most frequently identified.

The receptor tyrosine kinase c-MET is involved in cell proliferation, apoptosis, and cell migration as well as in DNA repair^[10]^. In addition, c-MET has been implicated in the regulation of the inflamed tumour microenvironment (TME), thereby contributing to an increased immune escape of tumour cells from T cell killing.

Several lines of evidence have demonstrated a clear relationship between c-MET alterations and the immune system; however, the underlying mechanisms are multifactorial and remain unclear. However, evidence exists that c-MET alterations are positively correlated with enhanced expression of immune-inhibitory molecules (e.g., PD-L1) and decreased expression of co-stimulatory markers (e.g., CD137, CD252), suggesting a putative role for immunotherapies after EGFR TKI treatment of NSCLCs harbouring MET alterations^[24,25]^.

Moreover, c-MET is generally accepted to be the major resistance mechanism following treatment with third-generation EGFR TKIs and is associated with a dismal prognosis in NSCLC patients. Although MET amplification was found in only 2%-5% of NSCLCs, MET amplification is a potential resistance pattern to EGFR TKIs in NSCLC. In osimertinib resistance, c-MET amplification is the most common off-target bypass mechanism, occurring in approximately 15% to 19% of patients depending on the treatment line and testing method. Additionally, high c-MET protein overexpression (detected via immunohistochemistry) is seen in 30% to 37% of advanced EGFR-mutated NSCLC cases post-osimertinib^[26-28]^.

Based on the preclinical and clinical studies, two highly specific c-MET inhibitors have been developed (capmatinib and tepotinib) which are approved for NSCLC patients harbouring c-MET amplifications or exon 14 skipping mutations^[29]^.

The proto-oncogene HER2 has been found to be mutated or amplified in many cancers, including breast, lung, and gastric cancer. Several groups of researchers have provided experimental evidence that mutated HER2 (mainly insertions in exon 20, 1%-2% of patients) can activate EGFR downstream signalling and thereby is involved in resistance to third-generation EGFR TKIs^[30]^.

Other major off-target resistance mechanisms responsible for failure of third-generation TKIs include oncogenic fusions (e.g., BRAF, FGFR, RET, and many others) which might be druggable by combination targeted therapy approaches^[16,31]^.

Beyond resistance mechanisms associated with changes in cellular morphology (e.g., histological changes, epithelial-mesenchymal transitions), many other EGFR-independent mutations confer resistance to osimertinib and other third-generation EGFR TKIs. Amongst them, mutations in the RAS/MEK/ERK and the PI3K/AKT/mTOR signal transduction pathways [Figure 2] are critical and can confer resistance to EGFR TKIs. Mutations in the (phosphoinositide-3-kinase) *PIK3CA* gene are of particular interest (NSCLC: 5%-8%)^[32]^. This mutation has been found to activate the PIK3CA/AKT/mTOR signal transduction cascade in various cancers, with the E545K alteration being the predominant *PI3KCA* gene mutation (detected in 10.5% of NSCLC patients harbouring PIK3-Cα alterations^[32]^). The PIK3CA-associated signalling pathway is involved in tumour cell proliferation, cell survival, and thereby can lead to EGFR TKI resistance.

Recently, Qiu *et al.* reported results from a retrospective analysis of NSCLC patients harbouring both *EGFR* and *PIK3CA* gene mutations^[33]^. They found that patients treated with EGFR TKIs had a significantly shorter mOS (20.6 *vs.* 32.4 months), supporting the proposal that NSCLC patients with PIK3CA mutations are clinically less responsive to EGFR TKIs.

Currently, PI3K inhibitors for tumours including NSCLCs with PIK3CA mutations are undergoing clinical evaluation. A summary of EGFR TKI resistance mechanisms is depicted in Figure 1.

## FOURTH-GENERATION EGFR TKIS - LIGHT AT THE HORIZON?

Resistance to first-generation (quinazoline-based, reversible inhibition) and second-generation (quinazoline-based, irreversible binding) EGFR TKIs is common in the clinic, with many patients relapsing after 9-14 months. The major cause of treatment failure is the development of the T790M mutation, which blocks binding to the protein’s ATP pocket.

The third-generation (pyrimidine-based, irreversible binding) TKI osimertinib, which is regarded as the standard of care for NSCLC patients harbouring EGFR mutations, has been found to be active against T790M; however, resistance will ultimately develop (known to be due to c-MET amplifications and/or C797S mutations) (reviewed by^[2]^). Several other off-target resistance mechanisms are also implicated [Figure 1]. The C797S mutation is more prevalent in osimertinib-resistant patients (6%-24% after first-line). The incidence of the acquired EGFR C797S mutation ranges from 10% to 26% (and up to 30% in some cohorts) following second-line osimertinib treatment for T790M-mutated NSCLC. It stands as the single most frequent on-target resistance mutation in this setting^[34]^.

The fourth-generation EGFR TKIs are orally bioavailable, and many of them, but not all, are allosteric thiazole amide-based reversible inhibitors^[35,36]^. Their activity results from selective binding to an allosteric site, which can alter EGFR protein conformation and, in turn, overcome the resistance mutation C797S^[36]^. Initially, they were designed as allosteric agents to overcome the C797S mutation (e.g., EAI045); the field has now broadened. Evidence suggests that fourth-generation EGFR TKIs include not only allosteric inhibitors but also ATP-competitive (e.g., BLU-945) and mixed allosteric/orthosteric inhibitors^[37]^. Orthoallosteric EGFR TKIs (e.g., FL30, 25g) are novel, fourth-generation inhibitors that simultaneously engage both the canonical orthosteric (ATP-binding) site and the allosteric pocket of the EGFR protein^[38]^. Of note, the IC_50_ for 25g (L858R/T790M/C797S) has been reported to be only 2.2 nM^[39]^; the IC_50_ for FL30 (L858R/T790M/C797S) was found to be 95 nM, which is comparable to that of osimertinib (87 nM)^[40]^.

In selected preclinical models, these novel compounds (either alone or in combination with other drugs) have demonstrated very potent activity, and many are now undergoing early clinical development, with silevertinib (formerly BDTX-1535) and HS-10504 being the most advanced drug candidates [Table 2] with impressive clinical activity. However, despite the potent efficacy of these novel fourth-generation EGFR TKIs in preclinical models, clinical development might be impeded by safety issues since these drugs are not mutation-specific, which could narrow the therapeutic window.

**Table 2:** Selected fourth-generation EGFR TKIs in clinical development

**Compound**	**Targets**	**IC_50_ for C797S**	**Phase**	**NCT number**	**Ref.**
Emupertinib (TAS-3351)	del19, L858R, T790M, C797S	< 10 nM	I	NCT05765734	Kasuga *et al.*^[41]^
H002	del19/L858R ± T790M ± C797S	NR	I/II	NCT05519293	Huang *et al.*^[42]^
TQB-3804	del19/T790M/C797S or L858R/T790M/C797S	0.13-0.46 nM	I	NCT04128083	Liu *et al.*^[43]^
QLH-11811	del19, L858R, T790M, C797S	2.6-50 nM	I/II	NCT05555212	Zheng *et al.*^[44]^
ES-072	L858R, T790M, C797S	0.74 µM	I	N/A	Zheng *et al.*^[45]^
NX-019	del19, L858R, exon20ins, C797S, G719S, L861Q	NR	I	NCT05514496	Wilson *et al.*^[46]^
BBT-176	del19/T790M/C797S or L858R/T790M/C797S	1.8-5.4 nM	I/II	NCT04820023	Lim *et al.*^[47]^
BBT-207	del19, L858R, T790M, C797S, L792H	< 10 nM	I/II	NCT05920135	Gawlic *et al.*^[48]^
HS-10504*	del19, L858R, T790M, C797S	NR	I/II	NCT06461156	Xie *et al.*^[49]^
JIN-A02	del19, L858R, T790M, C797S	61.5-84.4 nM	I/II	NCT05394831	Lee *et al.*^[50]^
WJ13404	del19/T790M/C797S or L858R/T790M/C797S	NR	I/II	NCT05662670	Wang *et al.*^[51]^
TRX-221	del19, L858R, T790M, C797X	2 nM	I/II	NCT06186076	Lim *et al.*^[52]^
BPI-361175	del19/T790M/C797S or L858R/T790M/C797S	15 and 34 nM	I/II	NCT05393466	Kadam and Patel^[53]^
STX-241	del19, L858R, C797S	0.3-1.7 nM	I/II	NCT06567015	Ruiter *et al.*^[54]^
EAI045	C797S	0.33 µM	Stopped	N/A	Jia *et al.*^[55]^
Silevertinib (BDTX-1535)	L858R, del19, T790M, L747P, L718X, E709X, S784F, V834L, A289V, C797S, exon20ins, EGFRvII-IV	< 10 nM	II/III	NCT05256290	Lucas *et al.*^[56]^

Data are derived from clinical trial registration information (NCT number: www.clinicaltrials.gov) and from references cited in the table. *Compound is already undergoing phase III evaluation (HS-10504 *vs.* platinum-doublet, second-line NSCLC, NCT 07754123). Trial is not yet open for recruitment. EGFR: Epidermal growth factor receptor; TKIs: tyrosine kinase inhibitors; NR: not reported; N/A: not applicable.

As already noted above, fourth-generation EGFR TKIs primarily target the ATP-binding pocket (specifically interacting with key catalytic residues like Lys745) or distinct allosteric sites outside the traditional kinase pocket^[57]^. Binding to the allosteric site leaves the ATP site accessible^[57]^, suggesting that there might be synergistic activity when administered together with ATP-competitive EGFR TKIs (early trials are ongoing to further investigate this proposal).

The race for novel EGFR TKIs will continue, and we can assume that the fifth generation will be on our doorsteps soon [Figure 1]. When adding pressure to a given biological system (e.g., EGFR TKIs and other drugs), the system will react: targeting the C797S resistance mutation is our development focus, however, other rare resistance mutations such as L718X (exon 18 with X = Q or V)^[58]^ might emerge as resistance mechanism following fourth-generation TKIs treatment in the future: Mother Nature has many arrows in the quiver.

Since the EGFR and the RAS/MEK/ERK pathways are closely intertwined [Figure 2], and mutations in the *MEK* and *ERK* genes are less commonly found, it is also conceivable that targets further downstream might be more attractive drug targets to overcome EGFR TKI resistance (e.g., atebimetinib or SKLB-D18).

To date, no fourth-generation EGFR TKI has been approved for clinical use, and initial results of the ongoing clinical trials are eagerly awaited. A selection of fourth-generation EGFR TKIs is summarized in Table 2.

## NOVEL RAS-MEK-ERK-INHIBITORS - NEW ARROWS IN THE QUIVER?

Over 20% of cancer patients are harbouring RAS mutations, with K-RAS mutations being the most frequent alterations with an incidence of more than 75% of all cases^[59]^. Historically, RAS oncogenes were considered undruggable, and initial attempts to target RAS with farnesyltransferase inhibitors were not promising (reviewed by^[60]^).

However, two irreversible Kirsten rat sarcoma virus (KRAS) inhibitors (targeting the G12C mutation), sotorasib (Lumykras®) and adagrasib (Krazati®), have recently been approved by the FDA and the EMA. Both drugs have shown impressive objective response rates (ORRs) in NSCLC (36% and 43%, respectively)^[61,62]^, but lower ORRs have been reported for colorectal carcinomas^[63]^.

Clinical trials combining EGFR inhibitors (e.g., cetuximab, amivantamab) with K-RAS G12C inhibitors (e.g., adagrasib, fulzerasib) in NSCLC have shown strong synergy. Designed to block adaptive feedback signalling that blunts K-RAS inhibitor monotherapy, these combinations have shown remarkable early-stage results, with ORRs up to 80% in first-line settings (reviewed by^[64]^). Moreover, early data from the KROCUS phase II trial (first-line G12C-mutated NSCLC, cetuximab plus K-RAS G12C inhibitor) demonstrated an ORR of 69% and a mPFS of 12.5 months^[65]^. In addition, clinical trials with amivantamab (bispecific monoclonal antibody, EGFR x c-MET) plus K-RAS inhibitors (e.g., olomorasib) are ongoing (NCT07227025; KaRAnaSa Trial). Studies targeting downstream effectors (SOS1 or SHP2, Figure 2) are also underway to more comprehensively block the K-RAS signal transduction cascade. In this regard, for the KontRASt-01 trial (NCT04699188, TNO155 [batoprotafib] plus JDQ443 [K-RAS G12C inhibitor]), an ORR of 90% was reported for treatment-naïve NSCLC patients harbouring G12C mutations (*N* = 12)^[66]^.

These response rates for NSCLC patients harbouring K-RAS mutations, together with a better understanding of the underlying molecular biology of the RAS-RAF-MEK-ERK signal transduction cascade, have prompted many research groups to develop new and more potent K-RAS inhibitors, which have now paved the way for a new generation of so-called pan-RAS inhibitors using the tri-complex inhibitor modality (e.g., daraxonrasib, formerly RMC-6236)^[67]^.

Daraxonrasib monotherapy has demonstrated impressive clinical results in second-line metastatic pancreatic carcinomas (mPFS 8.5 months, mOS 13.1 months, grade ≥ 3 adverse events: 65%)^[68]^, and FDA and EU approval is expected soon. In addition, as first-line therapy in combination with chemotherapy, an ORR of 58% and an OS at 6 months of 90% were reported; however, this was associated with a grade ≥ 3 toxicity rate of over 70%^[69]^, suggesting that safety may limit its future development. Nevertheless, a phase III trial in first-line is ongoing (RASolute-303, NCT05379985). A phase III trial with daraxonrasib monotherapy *vs.* docetaxel in K-RAS mutant NSCLC patients (RASolve 301, ISRCTN1793625) is also currently recruiting patients.

Zoldonrasib, selectively targeting the G12D mutation, has shown promising results in NSCLC patients in a phase I trial (ORR 52%, mPFS 11.1 months, OS at 12 months: 73%) (second-line, NCT06162221)^[70]^. A phase III trial (first-line) is planned (RASolve-308). However, fewer than 4% of NSCLC patients harbour G12D mutations^[71]^.

Combining fourth-generation EGFR TKIs with K-RAS inhibitors is a highly active research area aimed at overcoming tumour resistance in NSCLCs driven by EGFR and K-RAS mutations. This strategy targets adaptive signalling “bypass” loops that cancer cells utilize to maintain uncontrolled proliferation, suggesting that simultaneously blocking the initial driver mutation with a TKI and the bypass pathway with a K-RAS inhibitor might yield significant clinical benefit. Preclinical data show that combining these targeted therapies successfully suppressed the mutant RAS-ERK pathway^[72]^, and early clinical trials are currently planned.

Collectively, as of March 2026 (cutoff), 73 K-RAS inhibitors have entered 236 clinical trials evaluating essentially all K-RAS mutations. However, resistance develops rapidly, and toxicity may remain a significant issue (e.g., daraxonrasib). Safety concerns have now sparked considerable interest of researchers to target the downstream cascade of RAS signal transduction (e.g., MEK, ERK). This strategy was fueled by the findings that targeting MEK in tumours with secondary *cis* mutations is feasible in K-RAS-mutated cancers. Moreover, RAF inhibitors were found to activate RAF in K-RAS mutant cells (so-called “RAF inhibitor paradox”)^[73]^. Binding of a RAF inhibitor to a RAF monomer induces a conformational change that promotes binding of an adjacent non-inhibited RAF monomer, creating a catalytically active RAF dimer.

As MEK is a downstream target of RAS-RAF, novel MEK and ERK inhibitors are clearly warranted, since these compounds may be less susceptible to resistance mechanisms implicated in resistance to K-RAS inhibitors [Figure 2].

In an earlier study with binimetinib plus erlotinib (NCT01859026, phase I/Ib) in NSCLC patients harbouring activating K-RAS or EGFR mutations (*N* = 43) the ORRs were reported to be 5% (K-RAS-mutated patients) and 89% for EGFR-mutated treatment-naïve patients^[74]^. The safety profile was manageable; however, the clinical benefit of this combination was very modest, and the authors concluded that the development of novel EGFR and mitogen-activated protein kinase (MAPK) pathway inhibitors with better therapeutic indices is urgently warranted.

In this regard, atebimetinib is a novel, highly selective, oral, deep cyclic MEK inhibitor currently being evaluated in clinical trials as first-line treatment in combination with gemcitabine/nab-paclitaxel in metastatic pancreatic carcinomas (phase III, MAPKeeper-301).

In a phase I/II trial (first-line in metastatic pancreatic cancer in combination with gemcitabine and nab-paclitaxel), the compound has shown a much better safety profile (grade ≥ 3 adverse events: 0%), together with an even greater efficacy when compared with the pan-RAS inhibitor daraxonrasib (ORR 39%, mPFS 8.3 months, mOS 17.3 months)^[75]^. It should be noted that atebimetinib has also shown anti-cachexia and anti-polyneuropathy properties. Future evaluation of atebimetinib in NSCLCs is planned^[75]^.

Targeting ERK1,2 has been a major challenge over the last decades and has led several pharmaceutical companies to discontinue development programs, as many compounds have been found to be very toxic in preclinical animal models and early clinical trials. As a result, no ERK1,2 inhibitor has been approved to date^[76]^. In addition, tumour cells can adapt to treatment with ERK1,2 inhibitors, leading to rapid development of resistance. On the other hand, ERKs are located at the end of the RAS signal transduction cascade and have a low mutation rate; the development of selective and active inhibitors has sparked considerable interest among many researchers. Most recently, a Chinese research group^[77]^ has provided the first evidence that the newly developed ERK1/2/5 inhibitor SKLB-D18 showed superior efficacy against triple-negative breast cancer cell lines *in vitro* and *in vivo*. Using the human cell lines MDA-MB-231 and MDA-MB-468, SKLB-D18 (mechanistically an autophagy agonist that can mitigate multidrug resistance) was found to induce superior *in vitro* and *in vivo* anti-tumour activity compared with other ERK1/2 inhibitors. Of note, IC_50_ values were reported to be 38.7 nM (ERK1), 40.1 nM (ERK2), and 59.7 nM (ERK5), with almost no activity against 380 other proteins tested^[77]^. Interestingly, SKLB-D18 was significantly less toxic than other ERK inhibitors, a finding that, if confirmed in future studies, may open new treatment opportunities for other RAS-associated cancers, such as malignant melanoma, colorectal carcinomas, and pancreatic cancers.

## FUTURE DIRECTIONS

Currently, a range of treatment strategies is being investigated in clinical trials in patients with osimertinib-resistant EGFR-mutated NSCLCs, including novel fourth-generation TKIs, RAS/MEK/ERK inhibitors, new immune checkpoint inhibitors, VEGFR inhibitors/monoclonal antibodies, and bispecific antibodies as well as antibody-drug conjugates (ADCs) (reviewed by^[1]^). In particular, ADCs as an innovative treatment strategy for NSCLC patients with resistance to third-generation TKIs have sparked huge interest among several clinical research groups^[78]^. In contrast to highly specific targeted therapy approaches, ADCs have the potential to benefit a much broader patient group because these antibodies can address heterogeneous resistance mechanisms (i.e., EGFR-independent) that cause failure of third-generation TKIs. Some of these strategies (e.g., TKI-chemotherapy combination; bispecific monoclonal antibodies raised against c-MET and other targets) were found to have the potential to improve the efficacy of osimertinib, as they have demonstrated a trend toward better overall survival and a more beneficial therapeutic index, suggesting that these trials may serve as a “reference benchmark” for the treatment of osimertinib-resistant NSCLC patients. However, based on the currently available data, it is too early to draw definitive conclusions, and more preclinical and clinical work is needed.

Although we treat NSCLC patients in the era of precision oncology, a concept that requires personalised therapies, we cannot recommend an optimal treatment strategy and sequence for NSCLC patients with acquired EGFR TKI-resistant tumours. Therefore, an improved understanding of the underlying mechanisms of resistance in NSCLC will clearly help to pave the way for the development of innovative, individualised, and highly specific drugs for the therapy of osimertinib-resistant NSCLCs.

To tackle osimertinib resistance (e.g., C797S, c-MET) in NSCLC patients, much emphasis has been placed on developing novel fourth-generation EGFR TKIs, which have shown promising activity in earlier clinical trials^[79]^. However, targeting on-target resistance mechanisms alone might not be sufficient since other off-target pathways may also be operative in these cancers [Figures 1 and 2]. It rapidly became apparent that the best strategy for integrating future fourth-generation EGFR TKIs into the treatment armamentarium for NSCLC is unclear and warrants further preclinical and clinical research.

Novel SOS1 inhibitors (e.g., BI 1701963) block the interaction between SOS1 and RAS to suppress oncogenic signalling. They are currently evaluated in phase I/II clinical trials for NSCLCs, usually in combination, but have not entered phase III development as a standalone treatment strategy for lung cancer. Moreover, there are currently no active late-phase clinical trials evaluating SHP2 inhibitors specifically for NSCLCs (mostly due to toxicity-related issues). Innovative SHP2 inhibitors (e.g., RMC-4630 or JAB-3312) are primarily being investigated in early-phase trials for NSCLCs and K-RAS-driven tumours, usually combined with K-RAS or MEK/ERK inhibitors^[80,81]^.

Therefore, the provocative question remains whether novel fourth-generation TKIs may provide a significant benefit over currently available NSCLC strategies in terms of efficacy and patient outcomes. Given the low mutation prevalence of the C797S resistance mutation (up to 15%)^[82]^ in osimertinib-resistant NSCLC patients, development of these new compounds might be slow, particularly in the Western world, where only 10%-15% of all NSCLC patients are eligible for EGFR TKI therapies. Therefore, this would require identifying a small and specific patient population that would benefit most from this new class of agents. This also supports the assumption that demonstrating the superiority of fourth-generation TKIs in terms of clinical benefit will be associated with higher drug development difficulties/risks/costs.

Collectively, given the early clinical development stage (the vast majority of these compounds are in phase I/II evaluation) and the fact that only results from heavily pretreated patients have been presented so far, the putative clinical relevance of fourth-generation EGFR TKIs for NSCLC patients with progression after osimertinib still needs to be defined. The finding that many drug candidates of the fourth-generation EGFR TKIs target not only resistance mutations such as C797S, but also common mutations (e.g., del19, L858R) and compound mutations could be of great importance for NSCLC patients and may pave the way for more sophisticated EGFRmut treatment strategies; however, many challenging development hurdles need to be cleared before victory can be declared.

## DECLARATIONS

### Copyright

© The Author(s) 2026.
